# Clinical Significance of Circulating Cell-Free DNA Detection in Multiple Myeloma: A Meta-Analysis

**DOI:** 10.3389/fonc.2022.852573

**Published:** 2022-02-18

**Authors:** Xueshi Ye, Wanli Li, Lifei Zhang, Junyao Yu

**Affiliations:** ^1^ Department of Hematology, Sir Run Run Shaw Hospital, School of Medicine, Zhejiang University, Hangzhou, China; ^2^ Department of Orthopedics, the Second Affiliated Hospital, School of Medicine, Zhejiang University, Hangzhou, China

**Keywords:** circulating tumor DNA, cell-free DNA, multiple myeloma, minimal residual disease, prognosis, meta-analysis

## Abstract

Circulating cell-free DNA (cfDNA) detection, a non-invasive method, appears promising for genetic analyses as well as quantitative assessment of tumor burden in patients with cancer. Although the analysis of cfDNA for clinical prognosis and monitoring disease burden in multiple myeloma (MM) has been recently studied, the results are unclear. In this meta-analysis, we explored the clinical significance of circulating cfDNA detection in patients with MM. We searched PubMed, Embase, and the Cochrane Library for eligible studies published up until July 25, 2021. Diagnostic accuracy variables were calculated and analyzed using Meta-Disc, and prognostic data were analyzed using Review Manager. Overall, seven studies comprising 235 myeloma patients met our inclusion criteria. The overall sensitivity and specificity of cfDNA to detect minimal residual disease (MRD) were 0.58 and 0.91, respectively. Moreover, higher levels of cfDNA were associated with worse progression-free survival as well as with poor overall survival. Our meta-analysis revealed that ctDNA detection has an obvious advantage in terms of MRD detection specificity, but it showed no superiority over bone marrow assessment in terms of MRD detection sensitivity, and higher levels of cfDNA were indicative of worse prognosis in patients with MM. cfDNA detection is a non-invasive method and thus shows promise as a good alternative to BM biopsies for monitoring clonal evolution and tumor burden so as to guide the treatment of patients with MM.

## Introduction

Multiple myeloma (MM), an incurable hematological malignancy, is characterized by recurrent cytogenetic and molecular abnormalities. Malignant plasma cells show typical multifocal distribution in the bone marrow (BM) and occasional extramedullary dissemination (1). Considering the spatial heterogeneity of myeloma, analyzing BM aspirates collected from a single site often does not provide comprehensive insights into the genetic profile of tumors. During disease progression, drug resistance and clonal evolution pose major issues (2, 3). Longitudinal evaluation of mutational landscape and tracking tumor burden can facilitate the identification of early signs of treatment resistance and relapse. However, repeated BM aspiration is impractical as the procedure is invasive and causes discomfort. Although BM biopsy is the current gold standard for MM diagnosis and prognostic stratification, tracking malignant clones remains challenging because of the spatial and temporal limitations of BM biopsies; therefore, there exists an urgent need to identify a novel biomarker to monitor disease progression.

The presence of cell-free nucleic acid fragments in human blood was first described in 1948 by Mandel and Métais (4). Cell-free DNA (cfDNA) is believed to have originated as a consequence of cell apoptosis and necrosis, and possibly also active secretion (5). Increased cfDNA levels were first reported in the serum of cancer patients in 1977 (6). According to several studies, cfDNA is of potential diagnostic and prognostic importance in various cancer types, and its levels during treatment are reportedly correlated with outcome (7–9). With the development of molecular methods, mutant DNA fragments, confirmed to be of tumor origin, have been detected in plasma. Mutations in cfDNA can serve as highly specific markers for cancer, and tumor-derived DNA in cfDNA, also known as circulating tumor DNA (ctDNA) (10), detection provides a non-invasive approach to diagnose cancers. ctDNA carries information pertaining to the dynamics of cancer-specific genetic and epigenetic alterations; moreover, ctDNA represents the entire epitome of mutations present in primary and metastatic tumors (11). In comparison to previously used blood-based biomarkers, cfDNA-based detection methods show higher sensitivity, and thus, they seem to have great potential for both quantitative analysis of tumor burden and genetic analysis in case of patients with cancer.

Relative to other cancer types, cfDNA concentration is higher in MM, and there exists high concordance between mutations found in DNA using BM aspirates and those found in ctDNA (12, 13). Although cfDNA analysis for clinical prognosis and to monitor disease burden in MM has been recently reported (14, 15), the results are unclear. Therefore, herein we performed a meta-analysis to systematically explore the clinical relevance of circulating cfDNA in patients with MM.

## Materials and Methods

### Data Source and Search Strategy

We searched for eligible studies in PubMed, Embase, and the Cochrane Library using the following keywords: “circulating DNA” OR “cell free DNA” OR “ctDNA” OR “cfDNA” OR “blood DNA” OR “plasma DNA” OR “serum DNA” OR “liquid biopsy” AND “myeloma” OR “plasmacytoma” OR “plasma cell neoplasms” OR “plasma cell dyscrasias”. All the data retrieved were updated to July 25, 2021.

### Study Selection

Two investigators independently reviewed all the titles and abstracts obtained on implementing our search strategy. We reviewed potentially relevant articles in full to ensure that they satisfied the following inclusion criteria: (1) clinical studies comprising patients with MM, (2) samples collected from the peripheral blood, (3) availability of information pertaining to the diagnostic and prognostic importance of cfDNA or ctDNA or ability to obtain such information from published data, and (4) clarity regarding techniques and target genes. The exclusion criteria were as follows: (1) reviews, conference abstracts, case reports, and non-English publications; (2) circulating viral DNA; (3) lack of outcomes; and (4) republished articles or samples.

### Data Extraction

All relevant studies were assessed by full-text review and those meeting the inclusion criteria were included in final analyses. Both investigators independently extracted the following data from each selected article: first author details, publication year, number of patients, cfDNA detection method, target genes, outcomes, and minimal residual disease (MRD) detection accuracy. If the eligible studies already reported the hazard ratio (HR) of the outcomes [progression-free survival (PFS) and overall survival (OS)] and 95% confidence interval (CI), then the data were directly extracted *via* full-text review. However, if this information was not available, then the HR was estimated using methods previously reported by Tierney et al. (16).

### Study Quality Assessment

The quality of diagnostic studies was assessed using the revised Quality Assessment of Diagnostic Accuracy Studies (QUADAS-2) criteria (17) (**Table 1**). Besides, the quality of prognostic studies was evaluated according to the Newcastle–Ottawa scale (21) (**Table 2**). Newcastle–Ottawa scale scores of more than five stars were considered to represent high quality.

**Table 1:** Assessment of the quality of diagnostic studies using the QUADAS-2 criteria.

Study	Risk of bias				Concerns about applicability		
	Patients Selection	Index Text	Reference Standard	Flow and Timing	Patients Selection	Index Text	Reference Standard
Mazzotti et al., (18)	U	L	L	L	L	L	L
Biancon et al., (19)	U	L	L	L	L	L	L
Vrabel et al., (20)	L	L	L	L	L	L	L

L, low risk of bias; H, high risk of bias; U, unclear risk of bias.

**Table 2:** Main characteristics of the included studies.

NO	Study	Number of patients	Detection method	Detection item	Outcome	Comparison	NOS
1	Mazzotti C, 2018 (18)	37	NGS	MRD (IGH, IGK, IGL rearrangements)			
2	Biancon G, 2018 (19)	22	NGS	MRD (IGH rearrangement)	PFS	the frequency of clonal IGH <4.7% vs. ≥4.7%	6
3	Vrabel D, 2019 (20)	12	ASO-qPCR	MRD (IGH rearrangements)			
4	Mithraprabhu S, 2019_1 (22)	20	NGS	KRAS, NRAS, CTNNB1, EGFR, TP53, PIK3CA, FOXL2, GNAS, BRAF	OS	FA (%) <1 vs. > 1	6
5	Mithraprabhu S, 2019_2 (23)	52	NGS	KRAS, NRAS, BRAF, TP53	OS	FA (%) <1 vs. > 1	6
6	Li Q, 2020 (24)	17	ddPCR	KRAS, NRAS, BRAF	OS	undetectable vs. detectable	6
7	Deshpande S, 2021 (25)	75	cfDNA quantification	Total cfDNA level	OS, PFS	cfDNA level ≤ 25.2 ng/ml vs. > 25.2 ng/ml	6

MRD, minimal residual disease; FA, fractional abundance.

### Statistical Analysis

Diagnostic accuracy variables, such as sensitivity, specificity, likelihood ratios [i.e., positive likelihood ratio and negative likelihood ratio], diagnostic odds ratio, and summary receiver operating characteristic(SROC) curve were calculated and analyzed using Meta-Disc v1.4. Sensitivity and specificity were defined as the proportion of MRD-positive and -negative patients identified *via* ctDNA detection in plasma among all patients confirmed to be MRD positive and negative, respectively, on BM assessment by multiparametric flow cytometry (MFC) or next-generation sequencing (NGS). The pooled HR and 95% CIs for PFS or OS were analyzed with Review Manager v5.4.1. The I^2^ statistic was used to quantify heterogeneity among the studies. I^2^ > 50% represented high heterogeneity, and a random effects model was accordingly used; if I^2^ was <50%, a fixed effects model was used for analyses.

## Results

### Study Selection

**Figure 1:**
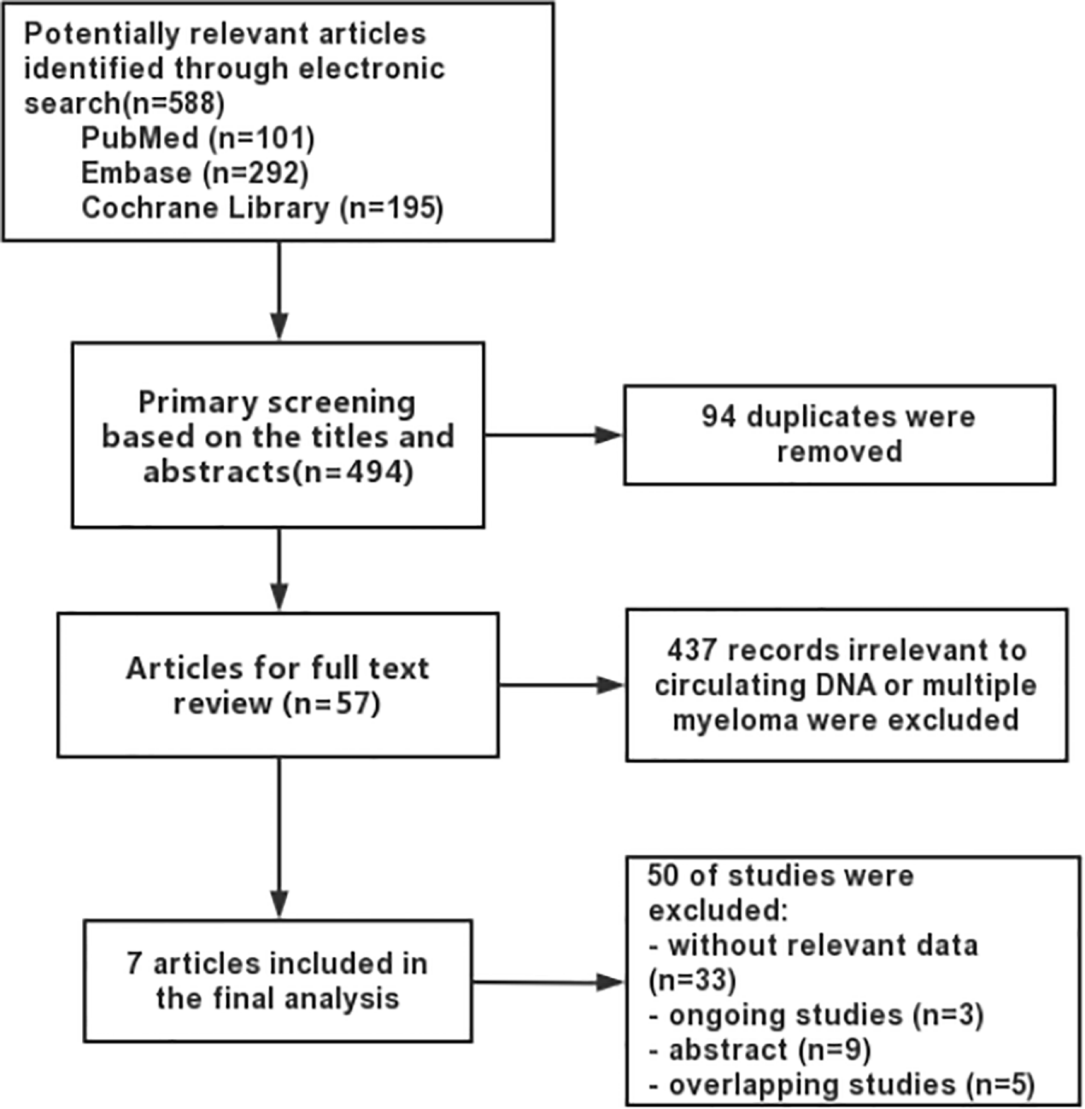
Flow diagram showing the selection of studies for meta-analysis.

Our search strategy led to the identification of 588 references, and 94 duplicates were removed (**Figure 1**). On screening the titles and abstracts, 57 articles were considered worthy of a thorough evaluation. Finally, after full-text review, seven studies comprising 235 myeloma patients were selected for our meta-analysis. Among them, 3 studies were available for calculating the overall sensitivity and specificity of MRD detection. They all used immunoglobulin gene rearrangements in cfDNA to track residual myeloma cells. These three studies used different ctDNA detection methods: Mazzotti (18) and Biancon (19) used NGS, while Vrabel (20) used ASO-qPCR. In addition to Biancon’s study (19) which explore the relationship between the tumor-associated IGH sequence and PFS, the other 4 articles (22–25) included analysis on the association of cfDNA and survival in patients with multiple myeloma. Except the levels of total cfDNA were quantified in Deshpande’s research, the other articles used NGS or PCR to detect the levels of tumor specific DNA. **Table 2** summarizes the main characteristics and quality assessment of the included prognostic studies.

### Relevance of cfDNA in MRD Detection in MM

**Figure 2:**
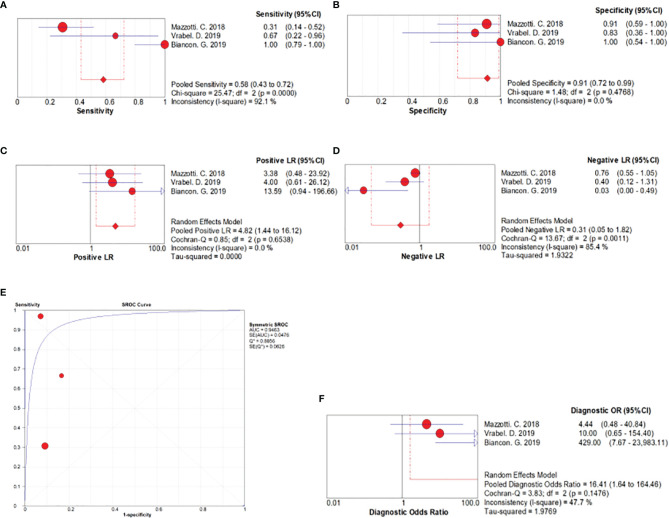
Forest plots related to MRD detection accuracy. **(A)** Forest plots for overall sensitivity, **(B)** overall specificity, **(C)** positive likelihood ratio, **(D)** negative likelihood ratio, **(E)** SROC curve, and **(F)** diagnostic odds ratio.

Three studies including 71 myeloma patients were pooled for the meta-analysis of MRD detection accuracy. As evident from **Figure 2**, the overall sensitivity and specificity were 0.58 (95% CI, 0.43–0.72) and 0.91 (95% CI, 0.72–0.99), respectively. The pooled positive and negative likelihood ratios were 4.82 (95% CI, 1.44–16.12) and 0.31 (95% CI, 0.05–1.82), respectively. The area under the SROC was 0.95, and the diagnostic odds ratio was 16.41 (95% CI, 1.64–164.46).

### Prognostic Significance of cfDNA in MM

**Figure 3:**
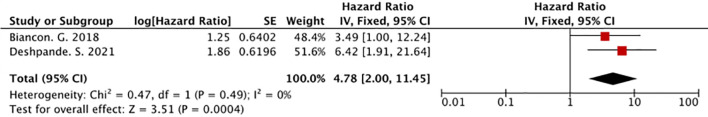
Forest plot showing the prognostic role of cfDNA on PFS.

**Figure 4:**
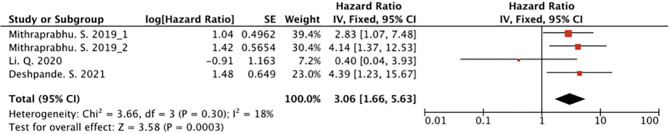
Forest plot showing the prognostic role of cfDNA on OS.

**Figure 5:**
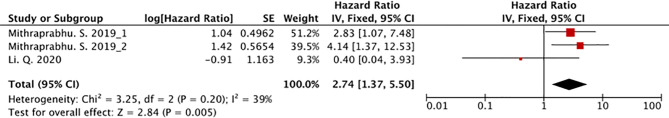
Forest plot showing the prognostic role of ctDNA on OS.

Five studies comprising 186 myeloma patients were pooled for the meta-analysis of survival. Of them, three articles included analysis on the association of cfDNA and OS in patients with MM (22–24), and one article included the information of both PFS and OS (25). Moreover, there was one study which only analyzed the association of cfDNA and PFS (19). In the two studies that included PFS as the outcome indicator, we found that high cfDNA levels in patients with MM were significantly associated with poor PFS (HR, 4.78; 95% CI, 2.00–11.45; P = 0.0004; **Figure 3**). We also found that high cfDNA levels in patients with MM were associated with worse OS (HR, 3.06; 95% CI, 1.66–5.63; P = 0.0003; **Figure 4**). To analyze the relationship between the level of tumor-derived DNA in circulating cfDNA and OS more specifically, we performed subgroup analysis using three studies that detected ctDNA levels. The results showed that high ctDNA levels in myeloma patients were associated with poor OS (HR, 2.74; 95% CI, 1.37–5.50; P = 0.005; **Figure 5**).

## Discussion

To date, myeloma remains an incurable disease, and patients often experience a relapse owing to residual tumor cells. MRD has become one of the most important biomarkers for outcome prediction and therapy optimization. However, longitudinal monitoring of BM MRD remains limited as repetitive BM biopsies are invasive to patients. In addition, in case of some patients with extramedullary disease, BM specimens may not accurately reflect the disease burden. Previous studies based on circulating cfDNA for MRD detection in various hematological malignancies have validated its significance for measuring a small number of residual tumor cells (26–28). Unfortunately, most such studies involving patients with MM either assessed only a few patients or lacking of the comparison with BM MRD assessment; therefore, the conclusions remain controversial.

Our pooled data confirmed that ctDNA detection had an obvious advantage in terms of MRD detection specificity (specificity, 0.91; 95% CI, 0.72–0.99). According to the guidelines for area under the SROC value interpretation (29), ctDNA presence in patients with MM has a relatively high diagnostic ability (AUC > 0.9) and can indicate MRD positive (**Figure 2**). Clonal immunoglobulin gene rearrangements in plasma samples have been detected by NGS or allele-specific oligonucleotide–qPCR to monitor tumor burden (18–20). At the time of MRD evaluation, the results of ctDNA detection using blood samples were compared with those of NGS or MFC using paired BM samples. Mazzotti (18) demonstrated the absence of a correlation between ctDNA and BM MRD detection by NGS using only immunoglobulin gene rearrangements in patients with MM. Further, Vrabel (20) compared ctDNA and BM MFC, a common method for MRD detection, and the sensitivity of MRD detection using ctDNA was only 66.7%. In contrast, Biancon (19) analyzed MRD by MFC using BM samples and found complete concordance with ctDNA data in all cases, and a high level of correlation was detected between ctDNA and BM MFC data (r = 0.5831, P = 0.0044, Pearson’s correlation test). Finally, regarding the sensitivity of MRD detection, ctDNA detection in plasma was not superior over BM assessment as per our analysis (sensitivity, 0.58; 95% CI, 0.43–0.72).

We believe that further studies with a larger cohort of patients achieving CR are warranted to determine the reasons for inconsistency in MRD detection sensitivity using ctDNA in different studies. The three studies pooled for the meta-analysis of MRD detection accuracy all used immunoglobulin gene rearrangements in cfDNA to track residual myeloma cells. However, MM is characterized by multiple recurrent somatic mutations, copy number variants, and structural alterations (30). Thus, given the extensive heterogeneity in MM, a large targeted sequencing panel may prove useful for improving MRD detection sensitivity using ctDNA.

In other cancers, cfDNA analysis has been used to identify mutations related to drug resistance and to predict therapeutic response, which could influence the choice of treatment (31–33). The prognostic value of cfDNA has been explored in myeloma. Although cfDNA is an admixture of normal and tumor-derived DNA (ctDNA), increased concentration of cfDNA signifies higher tumor burden and is indicative of the prognostic value of survival in many cancers (34, 35). In addition, high cfDNA levels at baseline are significantly associated with poor PFS and OS in patients with myeloma (25). ctDNA represents the entire epitome of mutations present in primary as well as metastatic tumors (36). Plasma-derived ctDNA analysis in patients with MM was found to show good concordance with standard mutation analysis using BM samples. Mithraprabhu (22, 23) analyzed plasma-derived ctDNA as an adjunct to BM biopsy for mutational characterization and tracking disease progression and found that in comparison with BM samples, plasma samples showed a higher proportion of TP53 mutations. Besides, Li (24) found a significantly higher detection rate of BRAF, KRAS, and NRAS mutations in plasma-derived cfDNA samples than in BM samples (53% vs 34%). Plasma evidently reflects mutations originating from all focal sites, both at the intramedullary and extramedullary levels. Therefore, assessing both BM and plasma samples should provide a more comprehensive landscape of tumor mutational burden.

The results of the studies included herein indicate the prognostic value of circulating cfDNA. cfDNA levels in MM are reportedly significantly associated with both PFS and OS. In two studies including PFS as the outcome indicator, higher levels of cfDNA were found to be associated with poor PFS (**Figure 3**) (19, 25). As evident from **Figure 4**, higher level of cfDNA was found to be associated with poorer OS. Because ctDNA only accounts for a small proportion of total cfDNA, we also performed subgroup analysis to assess the usefulness of ctDNA quantification to predict OS; we found that ctDNA-positive patients or those with a high level of ctDNA showed inferior OS (**Figure 5**).

In the analyzed studies, gene mutations, mainly including RAS-RAF and TP53, were detected, which may predict poor prognosis in patients with MM (37). Mithraprabhu (23) reported that high levels of ctDNA are a prognostic factor in case of relapsed/refractory MM patients but not in case of newly diagnosed MM patients. This could be because a higher frequency of gene mutations in plasma was detected in relapsed/refractory MM patients than in newly diagnosed MM patients. However, Li found a significantly higher detection rate of BRAF, KRAS, and NRAS mutations on using plasma-derived cfDNA samples (53%) than on using BM samples (34%) in case of newly diagnosed MM patients, and patients with these mutations showed shorter OS than those without them (24). These two studies used different ctDNA detection methods: Mithraprabhu used NGS, while Li used droplet digital PCR. Although previous studies have verified that these methods show high concordance in terms of tumor genotype (38, 39), the inconsistency in the data reported by Mithraprabhu and Li suggest that further studies are warranted to compare these methods; furthermore, more patients need to be evaluated to confirm the prognostic significance of plasma-derived ctDNA in newly diagnosed MM patients.

This study has several limitations that need to be addressed. First, the lack of currently recognized ctDNA gene targets in patients with MM might contribute to bias. Second, different ctDNA detection methods were used in the included studies. Finally, owing to the limited number of studies on ctDNA detection in MM, the data included is not very rich, which may contribute to further bias.

## Conclusions

To the best of our knowledge, this meta-analysis represents the first comprehensive study to investigate MRD detection and prognostic value of circulating cfDNA in patients with MM. We report that ctDNA detection has an obvious advantage in terms of MRD detection specificity; moreover, higher levels of cfDNA were found to be associated with worse prognosis in patients with MM. cfDNA detection is a non-invasive method and thus shows promise as a good alternative to BM biopsies for monitoring clonal evolution and tumor burden so as to guide the treatment of patients with MM. However, before its wide application in patients with MM, accurate ctDNA gene targets and standardized detection methods need to be established.

## Data Availability Statement

The original contributions presented in the study are included in the article/supplementary material. Further inquiries can be directed to the corresponding author.

## Author Contributions

XY conceived and designed the study. XY and WL collected and analyzed the data. LZ and JY analyzed the data. XY wrote the manuscript. All authors read and approved the final manuscript.

## Conflict of Interest

The authors declare that the research was conducted in the absence of any commercial or financial relationships that could be construed as a potential conflict of interest.

## Publisher’s Note

All claims expressed in this article are solely those of the authors and do not necessarily represent those of their affiliated organizations, or those of the publisher, the editors and the reviewers. Any product that may be evaluated in this article, or claim that may be made by its manufacturer, is not guaranteed or endorsed by the publisher.
